# Longitudinal association between body mass index and physical activity among adolescents with different parental risk: a parallel latent growth curve modeling approach

**DOI:** 10.1186/s12966-020-00961-4

**Published:** 2020-05-11

**Authors:** Parisa Naseri, Parisa Amiri, Somayeh Momenyan, Farid Zayeri, Mehrdad Karimi, Fereidoun Azizi

**Affiliations:** 1 Research Center for Social Determinants of Health, Research Institute for Endocrine Sciences, Shahid Beheshti University of Medical Sciences, Tehran, Iran; 2 Department of Biostatistics and Epidemiology, Qom University of Medical Sciences, Qom, Iran; 3 Proteomics Research Center and Department of Biostatistics, School of Allied Medical Sciences, Shahid Beheshti University of Medical Sciences, Tehran, Iran; 4 Department of Epidemiology and Biostatistics, School of Public Health, Tehran University of Medical Sciences, Tehran, Iran; 5 Endocrine Research Center, Research Institute for Endocrine Sciences, Shahid Beheshti University of Medical Sciences, Tehran, Iran

**Keywords:** BMI, Physical activity, Parental clusters, Parallel latent growth curve modeling

## Abstract

**Background:**

Data available on the association between physical activity (PA) and body mass index (BMI) in different periods of life is controversial. Using a parallel latent growth curve modeling (LGCM) approach, the current study aimed to investigate the influence of daily PA on adolescents’ BMI over a 12 year follow-up, taking into account their parental risk.

**Method:**

Participants comprised 1323 adolescents (53.5% girls), aged 12–18 years who had participated in the baseline phase of Tehran Lipid and Glucose Study (TLGS) (2001–2003), and were followed for an average period of 12 years. Physical activity, including leisure time and occupational activities, was assessed using the reliable and validated Iranian version of the Modifiable Activity Questionnaire (MAQ). Weight and height were objectively measured in order to calculateBMI.Atwo-step cluster analysis was conducted to classify parents into two high- and low-risk clusters. Parallel LGCM was fitted to estimate cross-sectional, prospective and parallel associations, which assessed the longitudinal association between simultaneous changes in PA and BMI during the study period. Analyses were stratified by gender and parental clusters.

**Results:**

A rising trend of BMI per 3 years was observed in boys 1.39 kg.m^2^(95% CI; 1.32, 1.48) and girls 0.9 kg.m^2^(95% CI; 0.82, 0.98), as well as in the low risk 1.11 kg.m^2^(95% CI; 1.03, 1.18) and high-risk 1.12 kg.m^2^(95% CI; 1.03, 1.22) clusters. Moreover, a positive prospective association between PA at baseline and BMI change over the 12 year follow-up, was observed in adolescents in the low-risk parental cluster 0.27(95% CI; 0.14, 0.41) indicating that higher levels of PA at baseline may lead to greater BMI in adolescents over time. However, examining longitudinal parallel association between simultaneous changes of PA and BMI per 3 years revealed adverse associations for adolescents in the low-risk parental cluster − 0.07 (95% CI; − 0.13, − 0.01) and in boys − 0.06 (95% CI; − 0.11, − 0.01).

**Conclusion:**

Despite a positive prospective association between BMI and PA at baseline, there was a weak inverse parallel association between these variables over time, particularly in boys and adolescents with low parental risk. These findings imply the potential role of other influential factors indetermining adolescents’ weight status which need to be considered in the future plannings.

## Background

Obesity is a chronic, often progressive disorder whichis strongly related to type 2 diabetes, hypertension, and cardiovascular diseases (CVDs) [1]. Many developed and developing countries are affected by this public health problem and a large body of evidence exists on the growing trend in mean body mass index (BMI) [2], and the prevalence of obesity and overweight worldwide [3]. Global statistics show that the prevalence of obesity and overweight increased in adolescents boys from 8.1 to 12.9% and in girls from 8.4 to 13.4% during 1980–2013 [4]. Iran as a country located in the Middle East and North Africa (MENA) region reported a high prevalence of obesity in recent decades; the total prevalence of general and abdominal obesity was estimated to be 11.89 and 19.12%in 2012, respectively [5]. The prevalence of obesity among Iranian children and adolescents is also increasing. In this regard, a meta analysis conducted in Iran, revealed that the prevalence of obesity among adolescents aged 12–18 years increased from 5.55% in 1995–1999 to 7% in 2005–2010 [6].

Although a number of studies showed an inverse relationship between PA and adiposity in adults [6–10], corresponding data for youth is controversial. In this regard, some evidence revealed no significant association between PA levels and excessive weight gain in childhood [11]. Accordingly, studies conducted in Iran indicated that PA levels were not associated with BMI in school-aged children [12, 13]. However, further findings showed that PA is associated with significant changes in BMI among adolescents [14], particulary among teenage boys [15, 16]. On the other hand, limited longitudinal data on the adult population revealed a parallel association between PA and BMI over time [17, 18]. To clarify these inconsistencies, longitudinal studies investigating the relationship between PA and weight status with regards to the main influential factors seem necessary.

A broad range of factors including genetic, behavioral, parental and socio-environmental are involved in childhood obesity [19]. Moreover, it seems that family members play a pivotal role in modeling these habits in a shared physical andsocial environment [20]. Accordingly, in a study conducted among Iranian adolescents investigating the association between parental obesity and cardio-metabolic risk factors, researchers demonstrated that the risk of excess weight (OR: 1.30, 95%CI: 1.17–1.44), obesity (OR: 1.36, 95%CI: 1.18–1.59) and abdominal obesity (OR: 1.16, 95%CI: 1.05–1.29) are significantly higher in adolescents whose parents have excess weight, compared to their normal weight counterparts [21]. The effects of different parental factors such as metabolic syndrome and its components, lifestyle behaviors including diet and physical activity as well as educational level on the incidence of non-communicable diseases (NCDs) have also been investigated in previous studies [22–24]. On the other hand, several studies conducted in Iran and other parts of the world have investigated the potential role of parental characteristics in the weight gain of their children. Results from these studies show a higher prevalence of obesity in youth with lower socioeconomic backgrounds [20, 23, 25, 26]. In our literature review, we found no published literature on the cumulative effects of these factors; according to these results, to account for the cumulative effects of parental characteristics, clustering these factors plays an important role in identifying groups who are at higher risk of chronic diseases [27].

To address the aforementioned gaps and beyond behavioral determinants of childhood obesity, the current study for the first time, aimed to assess the association between PA levels and BMI in adolescents with different parental risks. To do this, a two-step cluster analysis for identifying parental risk clusters and age adjusted parallel latent growth curve modeling (LGCM) for estimating cross-sectional, prospective, and parallel associations between these variables will be applied.

## Methods

### Study design and population

This study used data from the TLGS, a population-based cohort investigation aiming to determine the prevalence of NCD risk factors in a representative sample of residents from district 13 of Tehran. The design of the TLGS included two main components: phase 1, a cross-sectional study of 15,005 participants (women and men, aged ≥3 years), recruited between1999–2001 (baseline) using a multistage cluster sampling technique, with prospective ongoing follow-up examinations conducted every 3 years for the next 12 years (2004–2016). All socio-demographic, behavioral, anthropometric and clinical data were collected through face-to-face interviews by trained interviewers. Details of the TLGS have previously been published elsewhere [28].

The current longitudinal analysis has been conducted on data from the second to sixth follow-up examinations of the TLGS. A total of 1459 adolescents (54.4% girls), aged 12–18 years, participated in the second phase of the TLGS between 2002 and 2005; of these, 88 and 48 cohort members were excluded due to missing informationon BMI at baseline and incomplete data on parental socio-behavioral and metabolic syndrome (phase 2). Thus the final analytic sample included data on 1323 individuals (53.5% girls), followed up for an average period of 12 years with at least 2 follow-up examinations. The participants’ age range were 13–24, 16–28, 19–31 and 22–34 in the third, fourth, fifth and sixth phases respectively.

The study protocol was approved by the ethics committee of the Research Institute for Endocrine Sciences (RIES) of Shahid Beheshti University of Medical Sciences. Written informed consent was obtained from all participants, based on which, information on sex, age, parental clusters, physical activity and BMI were analyzed.

### Measurements

Using a pretested questionnaire, trained interviewers collected data on age, sex and physical activity of adolescents in addition to parental socio-behavioral factors (including job status, level of education, smoking, body weight status and Metabolic equivalent tasks (METs).

Physical activity level, including leisure time and occupational activities, was assessed using a reliable and validated Iranian version of the Modifiable Activity Questionnaire (MAQ) [29]. For adolescents aged < 18 years, leisure time was used and for those who were aged ≥18 years, both leisure time and occupational activities were taken into account. Participants were asked to report the physical activities in which they had participated during the past 12 months, in addition specifying the frequency and duration for each activity identified. Each activity was weighted by its relative intensity, referred to as MET. One MET is set at 3.5 ml of oxygen consumed per kg of body weight per minute and represents the resting metabolic rate. For all activity levels, obtained MET was multiplied by the time spent at each level. MET-time from each level was added to total 24 h MET- time, representing the average daily level of PA. For analytical purposes, leisure time physical activity (LTPA) values were standardized as z-scores. Moreover, levels of physical activity were defined as low (MET < 600 min/wk), moderate (MET 600–2999 min/wk) and high (MET ≥3000 min/wk) [30]. Weight was measured using digital scales, in light clothing and without shoes rounded to the nearest 100 g. Height was measured using a tape meter stadiometer. BMI was calculated as weight in kilograms divided by height in square meters. To remove subjective errors, all measurements were recorded by the same study personnel.

### Definition of terms

High-risk and low-risk parental clusters were identified based on the parents’ socio-behavioral characteristics and METs. For this purpose, their education levels were defined as primary, secondary and higher. Job status was categorized into two sub categories of employed and unemployed. Metabolic syndrome in parents, aged > 18 years was defined based on the Joint Interim Statement (JIS) [31] as the presence of any three of the following five riskfactors: (1) Abdominal obesity with a waist circumference (WC) ≥90 cm for both genders, based on Iranian cut off values [32, 33]; (2) HDL-C < 50 mg/dL in women and < 40 in men or receiving medical treatment to reduce HDL-C; (3) elevated TG level ≥ 150 mg/dL or medical treatment for hypertriglyceridemia; (4) hypertension ≥130 mmHg systolic blood pressure or ≥ 85 mmHg diastolicblood pressure) or using anti hypertensive treatment in a patient with a history of hypertension and (5) elevated FBG ≥ 100 mg/dL or drug treatment for the condition.

A two-step cluster analysis was conducted to classify parents in the two different classes. Cluster numbers were determined using the Schwartz’s Bayesian Criterion (BIC) index. Cluster analysis is an exploratory tool applied in order to organize observations or cases into different subgroups of individuals. In this method, the similarity of cases within each cluster is maximized and similarity between groups is minimized. There are a number of clustering procedures including hierarchical, k-means, and two-step cluster analysis. The hierarchical, k-means clustering and two-step procedure cluster analysis are more appropriate for small, moderate and large datasets (1000 cases or more), respectively. In addition, when a mixture of continuous and categorical variables are available, two-step procedure cluster analysis is recommended. In the current study, to detect intrinsic differences between participants, a two-step cluster analysis was used for continuous and categorical variables. For separation of adolescents, we first identified the variables significantly related with BMI and then included these variables in the cluster analysis.

### Statistical analysis

All analyses were gender-specific and have been stratified by parental clusters (low- and high risk). Baseline characteristics of the adolescent participants were summarized as means ± SD and frequencies (percentages) for continuous and categorical variables, respectively. Continuous variables were compared between gender and parental clusters using the independent samples T-test.

**Fig. 1:**
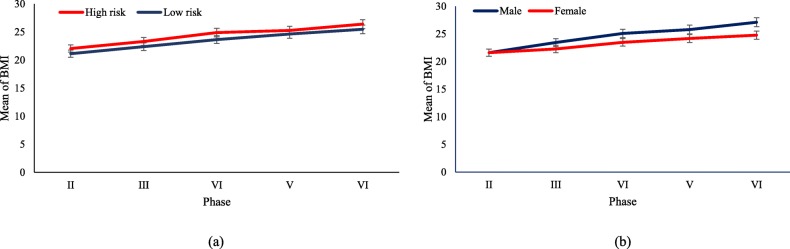
Trend of BMI of adolescents, aged 12–18 years: TLGS II- VI. **a** Based on parental clusters; P-interaction for parental clusters/year = 0.59. **b** Based on sex strata. P-interaction for sex/year< 0.001; Error bars shows standard error

**Fig. 2:**
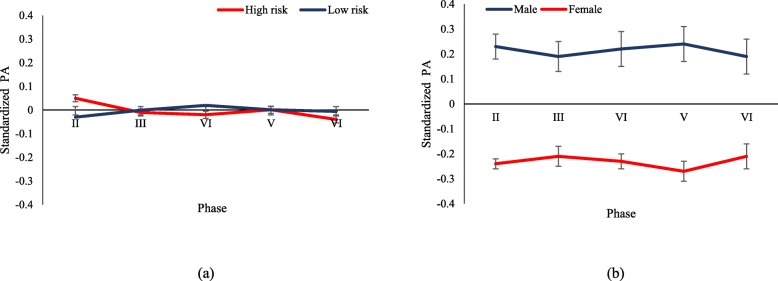
Trend of standardized physical activity (MET-minutes per week) of adolescents, aged 12–18 years: TLGS II- VI. **a** Based on parental clusters strata. P-interaction for parental clusters/year =0.51. **b** Based on sex strata. P-interaction for sex/year = 0.24; Error bars shows standard error

For descriptive purposes, mean BMI and standardized LTPA from the second phase (2004–2006) to the sixth phase (2014–2016) of the TLGS are illustrated in Figs. 1 and 2 by parental clusters and gender, separately. To assess the linear trend of BMI and standardized LTPA throughout the phases, a marginal model was fitted using the generalized estimating equation (GEE) methodology, separately for each category of parental cluster and gender. In each model, the time effect was reported as p for trend. In addition, differences in mean BMI and standardized LTPA were both examined over phases across the parental clusters and gender susing the interaction terms: parental clusters × phase and sex × phase.

**Fig. 3:**
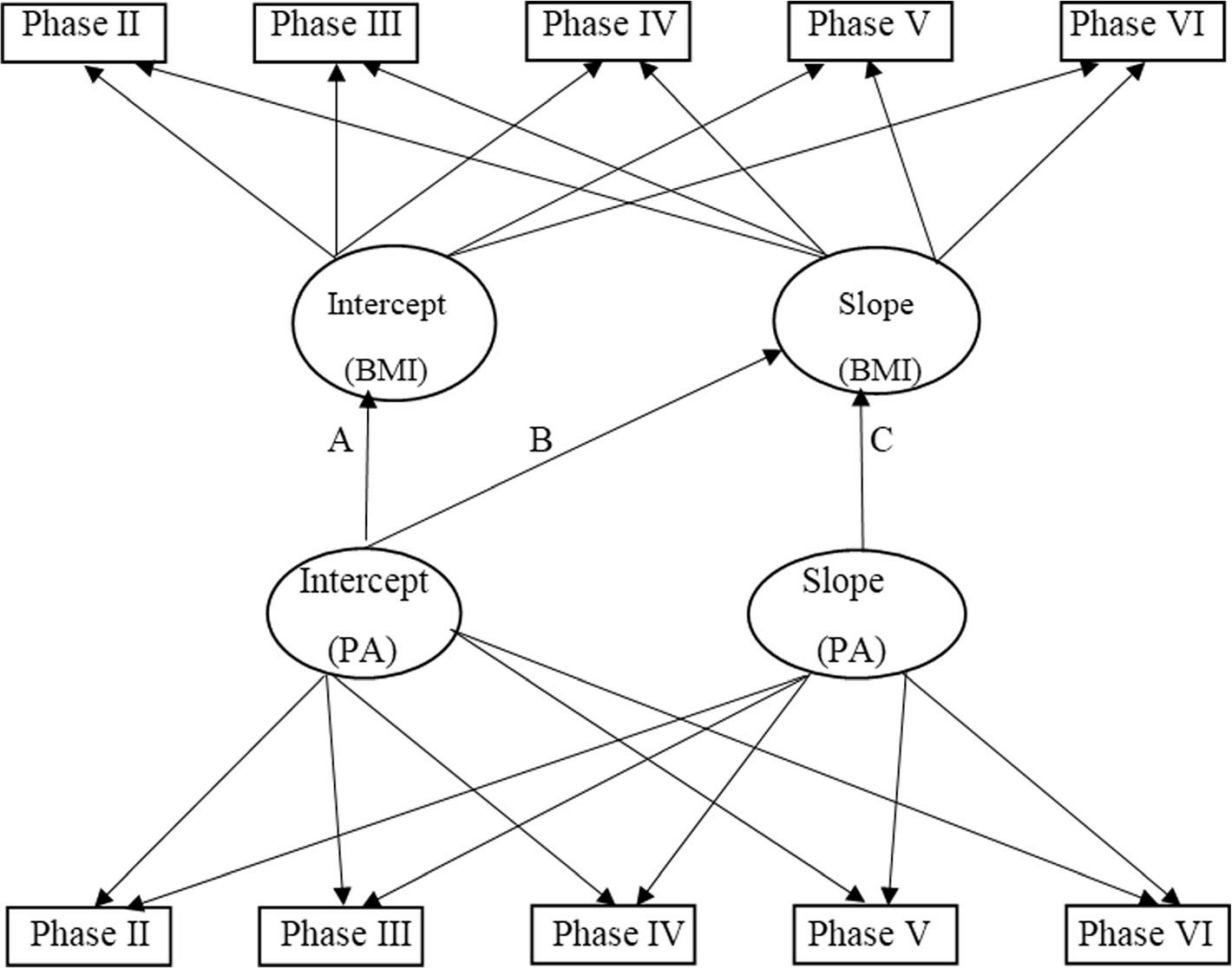
A schematic diagram depicting the parallel latent growth curve model; A: paths examining the cross-sectional associations of baseline PA with baseline BMI (Intercept (PA) → Intercept (BMI).B: paths examining the prospective associations of baseline PA with change in BMI (Intercept (PA) → Slope (BMI)). C: parallel associations of changes in PA with change in BMI (Slope (PA) → Slope (BMI))

In the analysis process, the longitudinal trajectories of BMI as well as standardized LTPA over the 12-year period were investigated using the LGCM, a multivariate statistical method applied within the structural equational modeling (SEM) framework to estimate growth patterns over time. Unconditional LGCM for outcome *y*_*ti*_ for individual *i* at time*t* is written as follows:

$$ {y}_{ti}={\uplambda}_{0t}{\upeta}_{0i}+{\uplambda}_{1t}{\upeta}_{1i}++{\upvarepsilon}_{ti}, $$

where η_0i_ = ν_0_ + z_0*i*_ and η_1*i*_ = ν_1_ + z_1*i*_. The outcome (*y*_*ti*_) can be predicted by two latent growth factors, η_0*i*_ (i.e., intercept) and η_1*i*_ (i.e., slope) and residual ε_*ti*_, in which ν_0_ and ν_1_ are latent means and z_0*i*_ and z_1*i*_ are individual deviations away from those means. In this analysis, a conditional LGCM for each outcome variable (BMI and standardized LTPA) was fitted and the latent growth factors of models were predicted using the baseline age covariate. To estimate the latent growth model parameters (intercept and slope), the factor loadings, λ_0*t*_ and λ_1*t*_, were constrained to [1] and [0, 1, 2, 3, 4], respectively.

Finally, a parallel LGCM was fitted to predict the latent growth factors of BMI, using the latent growth factors of the standardized LTPA (Fig. 3). This approach allows the linear relationship between PA and BMI to vary between individuals in both slope and intercept. In this model, some specific effects were assessed including: 1) a cross-sectional association between BMI and standardized LTPA at baseline; 2) a prospective association between baseline standardized LTPA and the change in BMI, and 3) parallel association between changes in standardized LTPA and change in BMI over the 12-year period.

The model goodness of fit was assessed using the comparative fit index (CFI), Tucker-Lewis index (TLI), and root mean square error of approximation (RMSEA). As atisfactory fit was accepted when CFI ≥ 0.9, TLI ≥ 0.9, and RMSEA≤0.08 [34]. The Mplus v7.2 (MutheÂn&MutheÂn, LA, CA), and IBM SPSS Statistics version 22 were used for data analysis and *p*-value < 0.05 was considered statistically significant.

## Results

**Fig. 4:**
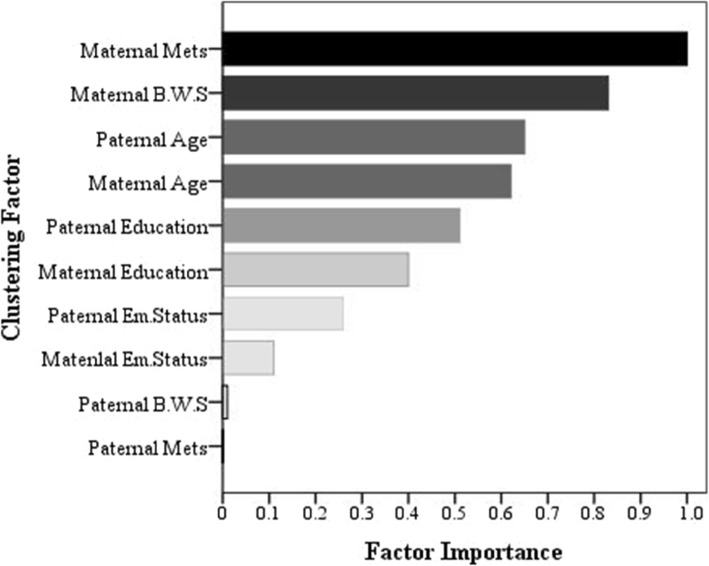
The importance of variables included in the cluster analysis

A total number of 1323 adolescents, aged 12–18 years on whom we had complete information on parental factors and BMI at baseline, were included in the cluster analysis. Three different socio-behavioral factors of parents (age, educational level, occupation) as well as METs, and BMI status were considered for parental cluster analysis. The most and the least important factors were maternal METs (with an importance value of 1) and paternal METs, respectively (Fig. 4- Appendix).

Results of the cluster analysis revealed two distinct parental clusters, referred to as low- and high-risk families. Table 4 (see Appendix) compares parental socio-behavioral status and MetS in these clusters. All parental socio-behavioral variables and METs were statistically significant between the two clusters (*p* < 0.001) which indicated difference among individuals in the low-and high-risk parental clusters regarding the aforementioned variables. However, we found no significant difference between the two groups in terms of the least important factor, i.e. paternal METs. In the high risk cluster, most parents had primary level education (74.8% of mothers and 68% of fathers). Moreover, the mean of maternal age in the high-risk cluster was higher than that of mothers in the low-risk cluster (45.41 versus 39.39 years) and the mean of paternal age was 44.55 and 51.88 years in the high-and low-risk parental clusters, respectively. In the low- and high-risk clusters, most mothers were unemployed (87.7 and 97.9%, respectively) while the corresponding rates for fathers were 7 and 29.9%, respectively. Regarding body weight status, 71.1 and 15.6% of mothers were obese in high- and low-risk parental clusters, respectively.

Baseline characteristics of adolescent girls and boys according to their parental clusters are presented in Table 1; the mean age of the participants was 14.65 (SD = 1.85) and 15.24 (SD =1.80) for low- and high-risk parental clusters, respectively. In addition, mean standardized LTPA was − 0.03 (SD = 0.93) and 0.05 (SD = 1.09) in the low- and high-risk parental clusters, respectively. Mean BMI for participants in the high-risk parental cluster was higher than in the low-risk one, a difference that was statistically significant (22.05 versus 21.14, *P* < 0.05). Sex-specific details are also presented in Table 1. Moreover, distribution of physical activity levels at basline is shown in Table 5 of Appendix. Mean BMI and standardized LTPA for parental clusters and gender categories duringthe TLGS phases are illustrated in Figs. 1 and 2, respectively. As shown, there was a significant rising trend of mean BMI among both parental clusters and gender groups (*P* < 0.05), however no significant trend for standardized LTPA was observed in the low-risk (0.008 (95% CI; − 0.025, 0.04); *P* = 0.637) and high-risk parental clusters (− 0.021 (95% CI; − 0.056, 0.014); *P* = 0.246), or in boys (− 0.004 (95% CI; − 0.042, 0.034); *P* = 0.845) and girls (− 0.001 (95% CI;-0.029,0.027); *P* = 0.937). Neither was the interaction of parental clusters and phases significant in BMI nor in standardized LTPA. In addition, the interaction of sex and phases was statistically significant for BMI (− 0.54 (95% CI; − 0.68, − 0.41); *P* < 0.001), which showed differences in BMI changes between males and females over time, while this was not significant for standardized LTPA.

**Table 1:** Baseline characteristics of study sample by gender and parental clusters

	Parental clusters			Sex		
	Low risk(*n* = 748)	High risk(*n* = 575)	*P*-value	Male(*n* = 623)	Female(*n* = 700)	*P*-value
Age (year)	14.65 ± 1.85	15.24 ± 1.80	< 0.001	14.87 ± 1.87	14.94 ± 1.84	0.46
Standardized Physical activity	− 0.03 ± 0.93	0.05 0.05 ± 1.09	0.217	0.23 ± 1.19	−0.24 ± 0.66	< 0.001
Physical activity	1357.9210 ± 1645.86	1503.8921 ± 1941.08	0.217	1821.20 ± 2113.82	984.76 ± 1179.00	< 0.001
BMI	21.14 ± 4.04	22.05 ± 4.33	< 0.001	21.53 ± 4.47	21.54 ± 3.93	0.97

Model fit indices based on parental clusters, gender and total population are presented in Table 2; LGCMs were fitted to each outcome variable (BMI and standardized LTPA) and then a parallel LGCM was fitted to these variables concurrently. Model fit indices showed that the fitted models were acceptable across parental clusters, genders and the total population. After adjusting for baseline age, longitudinal changes in BMI and standardized LTPA were well identified over the study period (RMSEA ≤.08, CFI ≥ .90, and TLI ≥ .90).

**Table 2:** Model fit indices for the latent growth curve models by parental clusters and gender

	Outcome: BMI				Outcome: PA				Parallel LGCM			
	*χ*^2^/*df*	RMSEA (90% CI)	CFI	TLI	*χ*^2^/*df*	RMSEA (90% CI)	CFI	TLI	*χ*^2^/*df*	RMSEA (90% CI)	CFI	TLI
Low risk	73.46/10	0.08 (0.07–0.10)	0.96	0.96	32.70/10	0.05 (0.03–0.07)	0.77	0.77	155.99/41	0.059 (0.050–0.07)	0.95	0.94
Haigh risk	28.07/10	0.05 (0.03–0.07)	0.95	0.95	34.85/10	0.06 (0.04–0.09)	0.60	0.60	87.51/41	0.04 (0.03–0.05)	0.95	0.94
Male	46.55/10	0.07 (0.05–0.09)	0.92	0.92	27.54/10	0.05 (0.03–0.07)	0.74	0.74	118.59/41	0.053 (0.04–0.06)	0.93	0.92
Female	37.52/10	0.05 (0.04–0.08)	0.97	0.97	11.76/10	0.01 (0.00–0.04)	0.96	0.96	90.79/41	0.03 (0.02–0.05)	0.97	0.97
All	28.07/10	0.05 (0.03–0.07)	0.95	0.95	34.27/10	0.044 (0.02–0.06)	0.80	0.81	135.48/41	0.041 (0.03–0.05)	0.96	0.95

Table 3 presented parameter estimates for described LGCMs and the parallel LGCM. The latent growth parameters estimated from the LGCM of BMI demonstrated significant increases in BMI per 3 years (estimated slopes for all models) for both parental clusters; 1.11 kg.m^2^ (95% CI; 1.03, 1.18) and 1.12 kg.m^2^(95% CI; 1.03, 1.22) in the low- and high-risk parental clusters, respectively. These values were 1.39 kg.m^2^ (95% CI; 1.32, 1.48) in boys and 0.9 kg.m^2^ (95% CI; 0.82, 0.98) in girls and 1.12 kg.m^2^(95% CI; 1.06, 1.18) for the total population. However, the estimated slope for standardized LTPA across the five TLGS phases was not significant in the above-mentioned grouping variables. Moreover, there was no significant covariance between latent growth parameters of BMI and standardized LTPA based on parental clusters, gender groups and total population, indicating that there was no linear relationship between baseline BMI (standardized LTPA) and per 3 years changes in BMI (standardized LTPA) in these groups. Regarding the results of the parallel LGCM, baseline level of standardized LTPA was not significantly associated with BMI at baseline (effect of baseline standardized LTPA on baseline BMI) across parental clusters, gender strata or the total population. In other words, there was no significant cross-sectional association between LTPA and BMI at baseline. Meanwhile, a significant positive prospective association between baseline LTPA and per 3 years changes in BMI over the 12-year period (effect of baseline standardized LTPA on slope BMI) was observed in the low-risk parental cluster 0.27 (95% CI; 0.14, 0.41) and total population 0.18 (95% CI; 0.08, 0.28). After increasing LTPA at baseline, BMI increased by 0.27 in the low-risk parental cluster and 0.18 in the total population over time. We also found no significant associations in gender groups or in the high-risk parental cluster. Finally, a significant parallel association between changes in standardized LTPA and changes in BMI per 3 years (effect of standardized LTPA slope on BMI slope) was observed in the low-risk parental cluster − 0.07 (95% CI; − 0.13, − 0.01), boys − 0.06 (95% CI; − 0.11, − 0.01) and total population − 0.05 (95% CI; − 0.09, − 0.01). In other words, increasing LTPA over time led to decreasing BMI during the study period in the low-risk parental cluster as well as the total population.

**Table 3:** Estimated mean and 95% confidence intervals of latent growth parameters by parental clusters, gender and whole population

	Low risk		High risk		Male		Female		All population	
	Estimate	95% CI	Estimate	95% CI	Estimate	95% CI	Estimate	95% CI	Estimate	95% CI
**Outcome: BMI**										
Intercept _(BMI)_	21.27	(20.98, 21.55)	22.16	(21.81, 22.50)	21.78	(21.42, 22.13)	21.63	(21.35, 21.92)	21.65	(21.42, 21.87)
Slope _(BMI)_	1.11	(1.03, 1.18)	1.12	(1.03, 1.22)	1.39	(1.32, 1.48)	0.9	(0.82, 0.98)	1.12	(1.06, 1.18)
Cov_(BMI)_	−0.30	(−0.72, 0.11)	−0.17	(−0.76, 0.40)	−0.46	(−1.07, 0.13)	−0.19	(−0.58, 0.19)	−0.23	(−0.59, 0.12)
**Outcome: PA**										
Intercept _(PA)_	−0.04	(−0.11, − 0.00)	0.01	(− 0.08, 0.09)	0.21	(0.12, 0.29)	−0.24	(− 0.3, − 0.19)	0.72	(0.23, 1.22)
Slope _(PA)_	0.001	(−0.03, 0.02)	−0.02	(− 0.05, 0.01)	−0.01	(− 0.04, 0.02)	−0.004	(− 0.03, 0.02)	−0.05	(− 0.26, 0.15)
Cov _(PA)_	−0.05	(− 0.10, 0.03)	−0.01	(− 0.07, 0.05)	−0.04	(− 0.11, 0.03)	−0.03	(− 0.06, 0.01)	−0.03	(− 0.08, 0.01)
**Parallel**										
**Cross-sectional association**^**c**^										
Intercept _(PA)_ → intercept _(BMI)_	0.00	(−0.02, 0.02)	0.01	(−0.01, 0.03)	−0.01	(− 0.03, 0.007)	0.02	(− 0.01, 0.0.03)	0.00	(− 0.01, 0.02)
**Prospective association**^**d**^										
Intercept _(PA)_ →slope _(BMI)_	0.27	(0.14, 0.41)	0.08	(−0.07, 0.24)	0.09	(−0.07, 0.25)	0.08	(−0.03, 0.18)	0.18	(0.08, 0.28)
**Parallel association**^**e**^										
Slope _(PA)_→ slope _(BMI)_	−0.07	(−0.13, − 0.01)	−0.03	(− 0.08, 0.02)	−0.06	(− 0.11, − 0.01)	−0.04	(− 0.09, 0.005)	−0.05	(− 0.09, − 0.01)

The estimates are unstandardized regression coefficients; *P-value* < 0.05 is statistically significant

Time scale is per phase (every 3 years)

c: the estimates can be interpreted as changes in baseline BMI (kg_m2/phase) by each unit increase in baseline PA

d: the estimates can be interpreted as changes in the growth rate of BMI (kg_m2/phase) by each unit increasein baseline PA

e: the estimates can be interpreted as changes in the growth rate of BMI (kg_m2/phase) by each unit increasein growth rates of PA

## Discussion

This study aimed to examine cross-sectional and longitudinal associations between PA and BMI among TLGS adolescents with different parental risk. While there was no relationship between adolescents’ PA and BMI at baseline, our findings indicated a significant longitudinal association between these two variables in the low-risk cluster. Considering parallel associations between simultaneous changes in PA and BMI over time, the current findings revealed a positive effect of PA on decreasing BMI among male adolescents and those in the low-risk cluster.

In the current study, parental body weight status, occupation, education, age as well as METs in mothers, were the main discriminating variables at baseline between low- and high-risk parental clusters. Parents play a pivotal role in shaping offspring habits within a shared physical and social environment which could affect their lifestyle during childhood [20, 35]. Although the influence of parental role-modeling will decrease over time, it can be reasonable to assume that the effect7 will remain until adulthood. In the current study, on average both parents in high-risk clusters were older, less educated and more overweight/obese than their counterparts in the low-risk cluster, a finding that was consistent with those of previous studies [23, 24, 36]. Few studies have focused on the synergistic effects of parental factors on the BMI and lifestyle behaviors of children including PA, which makes it difficult to compare current results with those previously documented. Considering this limitation, current results regarding no significant difference in PA level between low- and high-risk parental clusters is in line with previous reports that also revealed no relationship between parents’socioeconomic status (SES) and PA levels in their offspring [37, 38]. Among SES factors, parents educational levels (unlike job status) were rarely associated with the PA of their children [39]. However, other findings from Iran also report no significant correlation between children’s PA level and parents’education as well as job status [40]. Instead of PA, our study indicated significantly higher BMI in adolescents classified in the high-risk parental cluster. A study conducted among Swedish children showed an inverse association between parental education and BMI [41]. In addition, other studies from Iran showed that children from lower SES families are estimated to be at significantly higher risk of becoming overweight/obese, compared to children whose parents also had higher mean BMI [21, 42–44]. In regard to the synergistic effects of parental factors, a study revealed higher risk for incidence of overweight in the high-risk cluster compared with those in the low-risk cluster [45].

In the current study, although boys were more active than girls, both genders had inadequate levels of PA. Studies across the world show different patterns of PA in girls and boys. While more sedentary recreational behaviors have been observed in boys, they were still more physically active than girls [46, 47]. Consistent with the current findings, other studies from Iran indicate higher levels of PA in boys than girls [12, 48–50]. In addition, the current findings showed no significant difference in mean BMI between girls and boys. These results are similar to those of a national survey of Iranian adolescents reporting that mean BMI in girls was higher than boys, this difference however was not statistically significant [51].

Based on our findings, there was no significant cross-sectional association between PA and BMI at baseline. This result may be related to nature of PA as a lifestyle component, it could be changed during life course [52, 53]. Accordingly, the effect of this behavior is revealed during the follow-up period rather than at baseline. Cross-sectional data from Iran and other countries showan inverse relationship between BMI and PA in adolescents [7, 54], however, another study showed no cross-sectional association between time spent on physical and sedentary activities with BMI-for-age among high school students in Tehran [12].

In the present study, significant longitudinal and parallel associations between adolescents’ BMI and PA were observed in the low-risk parental cluster, indicating that the more children are active at baseline and during follow-up years, the lower the increase in mean BMI over the study period. The current findings are in agreement with a previous longitudinal study from Europe indicating that higher levels of total PA and moderate-to-vigorous PA (MVPA) were associated with lower BMI in children over a 5 year follow-up [55]. In the current study, there were no significant longitudinal and parallel associations between adolescents’ BMI and PA in the high-risk parental cluster, which can be related to the effect of other variables such as adolescents’ dietary patterns, genetic and metabolic backgrounds. The association between parental obesity and metabolic disorders andoverweight in children have been well-documented [56, 57]. In this regard a study showed that schoolchildren from families with at least one parent with metabolic disorder had significantly higher BMI at baseline measurement and faster BMI progression during their growth period [58]. Accordingly, another study indicated that half of the offspring of mothers with type 2 diabetes weighed higher than the 90th percentile for their age population [59]. In addition to metabolic backgrounds, the important effectof children’s genetic backgrounds [60] as well as socio-behavioral factors including dietary habits and socio-economic status (SES) on adolescents’ weight status have been determined [42, 43]. In this regard previous studies revealed that low SES families mainly charachterized by lower income and education have little access to healthy foods and are more likely to consume high-calorie, low nutrient food stuffs [61, 62]. All these factors could modify the effect of PA levels on adolescent weight status inthe high-risk parental cluster.

The strength of the current study, is its consideration of parental data and its synergistic effect as an important factor in childhood obesity. Compared to previous studies, its 12 year follow-up could also be considered as a strength of the present study from Iran, a region of the Middle East. From an analytical point of view, our study applied a precise longitudinal method. Previous studies only considered changes of outcome variables between baseline and end of follow-up period while this analytical approach modeled observations in each phase of the study. In addition, all parameters related to cross-sectional, prospective, and parallel associations among variables were estimated. However, the results were affected by some limitations including unavailable data on adolescents’ diet as an important modifier. Moreover, it has been well-established that there may be biological and genetic factors that contribute to the variability of physical activity on body weight regulation which require further investigation [63, 64]. Furthermore, subjective methods were used to measure PA which could affect the accuracy of data by creating recall bias. Last but not least, it would have been useful to assess the association between PA and BMI, considering different body weight status at baseline to design related preventive programs and interventions.

## Conclusions

Our study showed a weak inverse parallel association between PA and BMI in adolescent boys and those with low-risk parents over the follow-up period. Similar results were not observed in adolescents with high parental risk and in girls. Current findings imply the pivotal role of other influential factors including metabolic and genetic backgrounds as well as SES and dietary patterns to determine adolescents’ weight status; these findings need to be considered in future plannings for weight management in the early years of life.

## Data Availability

The datasets used and/or analyzed during the current study are available from the corresponding authors on reasonable request.

## Appendix

**Table 4:** Descriptive statistics of parents’ socio-behavioral and MetS status in two clusters

	Parental clusters		
	Low risk (*n* = 748)	High risk (*n* = 575)	*P*-value
**Mothers’ characteristics**			
Age (years)	39.39 ± 4.94	45.41 ± 6.77	< 0.001
Level of education			< 0.001
Primary	271 (36.2%)	430 (74.8%)	
Secondary	410 (54.8%)	137 (23.8%)	
Higher	67 (9%)	8 (1.4%)	
Occupation			< 0.001
Employed	91 (12.2%)	12 (2.1%)	
Unemployed	657 (87.7%)	563 (97.9%)	
MetS status			< 0.001
Yes	133 (17.8%)	447 (77.7%)	
No	615 (82.2%)	128 (22.3%)	
Body weight status			< 0.001
Normal	186 (24.9%)	27 (4.7%)	
Overweight	445 (59.5%)	139 (24.2%)	
Obese	117 (15.6%)	409 (71.1%)	
**Fathers’ characteristics**			
Age (years)	44.55 ± 5.76	51.88 ± 8.15	< 0.001
Level of education			< 0.001
Primary	179 (23.9%)	391 (68.0%)	
Secondary	395 (52.8%)	114 (19.8%)	
Higher	174 (23.3%)	70 (12.2%)	
Occupation			< 0.001
Employed	696 (93%)	403 (70.1%)	
Unemployed	52 (7%)	172 (29.9%)	
MetS status			0.169
Yes	441 (59%)	323 (56.2%)	
No	307 (41%)	252 (43.8%)	
Body weight status			0.008
Normal	223 (29.8%)	207 (36%)	
Overweight	343 (45.9%)	264 (45.9%)	
Obese	182 (24.3%)	104 (18.1%)	

**Table 5:** Distribution of levels of physical activity based on parental clusters and gender

	Parental clusters			Sex		
	Low risk n(%)	High risk n(%)	*P*-value	Male n(%)	Female n(%)	*P*-value
**Physical activity levels**						
Low	428 (57.2)	330 (57.4)		269 (43.2)	489 (69.9)	
Moderate	267 (35.7)	191 (33.2)	0.25	274 (44)	184 (26.3)	< 0.001
High	53 (7.1)	54 (9.4)		80 (12.8)	27 (3.9)	

## Abbreviations

SES: Socioeconomic status
BMI: Body mass index
PA: Physical activity
LGCM: Latent growth curve modeling
TLGS: Tehran lipid and glucose study
MAQ: Modifiable Activity Questionnaire
CVDs: Cardiovascular diseases
MENA: Middle East and North Africa
NCD: Non-communicable disease
MetS: Metabolic syndrome
MET: Metabolic equivalent
LTPA: Leisure time physical activity
JIS: The Joint Interim Statement
WC: Waist circumference
BIC: Bayesian criterion
GEE: The generalized estimating equation
SEM: The structural equational modeling
CFI: The comparative fit index
TLI: Tucker-Lewis index
RMSEA: Root mean square error of approximation
